# Vitamin K Intake and Risk of Lung Cancer: The Japan Collaborative Cohort Study

**DOI:** 10.2188/jea.JE20220063

**Published:** 2023-10-05

**Authors:** Fangyu Yan, Ehab S. Eshak, Ahmed Arafa, Akiko Tamakoshi, Hiroyasu Iso, the JACC Study Group

**Affiliations:** 1 Public Health, Department of Social Medicine, Osaka University Graduate School of Medicine, Osaka, Japan; 2 Center for Surveillance, Immunization, and Epidemiologic Research, National Institute of Infectious Diseases, Tokyo, Japan; 3 Department of Public Health and Preventive Medicine, Faculty of Medicine, Minia University, Minia, Egypt; 4 Advanced Clinical Epidemiology, Medical Data Science, Osaka University Graduate School of Medicine, Osaka, Japan; 5 Public Health, School of Health, Calvin University, Michigan, USA; 6 Department of Public Health, Faculty of Medicine, Beni-Suef University, Beni-Suef, Egypt; 7 Department of Public Health, Graduate School of Medicine, Hokkaido University, Sapporo, Japan; 8 Institute for Global Health Policy Research, Bureau of International Health Cooperation, National Center for Global Health and Medicine, Tokyo, Japan

**Keywords:** vitamin K, lung cancer, prospective study, Japan

## Abstract

**Background:**

Limited reports from prospective human studies investigated the possible role of vitamin K in the development of lung cancer although vitamin K’s anticarcinogenic activities were verified from several in vitro and in vivo studies. We investigated the associations between total vitamin K intake from food and the development of lung cancer based on this large prospective cohort study.

**Methods:**

A validated food frequency questionnaire was used to examine vitamin K intake among 42,166 (16,341 men and 25,825 women) at the Japan Collaborative Cohort Study’s baseline (1988–1990). Hazard ratios (HRs) and 95% confidence intervals (CIs) of incident lung cancer were calculated using the Cox proportional hazard regression method based on vitamin K consumption quartiles.

**Results:**

430 cases (308 males and 122 women) of lung cancer were documented during a total of 564,127 person-years of follow-up (median follow-up, 14.6 years). Vitamin K consumption was shown to be inversely related to lung cancer risk; the multivariable hazard ratio [HR] for the highest versus lowest quartiles was 0.67 (95% confidence interval [CI], 0.46–0.96; *P* for trend = 0.010). This relationship appears to be stronger in males (HR 0.62; 95% CI, 0.40–0.96; *P* for trend = 0.016) than in females (HR 0.82; 95% CI, 0.42–1.61; *P* for trend = 0.39) (*P* for interaction = 0.012), and in ever smokers (HR 0.57; 95% CI, 0.36–0.91; *P* for trend = 0.006) than in never smokers (HR 0.79; 95% CI, 0.40–1.55; *P* for trend = 0.37) (*P* for interaction = 0.30). The individuals’ age, body mass index, or alcohol consumption status had no effect on the observed connection.

**Conclusion:**

Vitamin K consumption reduces the risk of lung cancer. More research is needed to clarify the molecular processes behind this connection.

## INTRODUCTION

With around 2.21 million new cases and 1.8 million deaths in 2020, lung cancer is a prominent source of morbidity and mortality globally^1^ Lung cancer claimed the lives of 53,208 men and 21,170 women in Japan in 2015,^2^ and it claimed the lives of 53,336 men and 22,055 women in 2019, which was the leading cancer site among men and the second leading among women.^3^ As a result, identifying modifiable risk factors that can reduce the burden of lung cancer is critical. In addition to smoking, well-established evidence showed that other modifiable lifestyles and nutritional factors may affect lung cancer risk.^4^^–^^6^ For example, the intake of vitamins A, C, and E, β-carotene, and fiber has been suggested to protect against lung cancer.^4^^,^^7^^,^^8^ Intakes of soy foods, dairy, and nuts were found to be related to decreased lung cancer risk,^4^^,^^8^^,^^9^ whereas red meat and coffee consumption were both linked to an increased risk.^10^^,^^11^

Vitamin K, also known as phylloquinone (vitamin K1) and menaquinones (vitamin K2), is a fat-soluble vitamin^12^ that may be found in the diet. Menaquinones are mostly found in fermented foods, such as fermented soy foods, pork, and dairy products, whereas phylloquinone is mostly found in green leafy vegetables.^13^ Vitamin K has been proven from in vitro and in vivo investigations to inhibit cell proliferation in a variety of cancer cell lines.^14^^–^^20^ Menadione (vitamin K3), a synthetic version of vitamin K having substantial growth inhibitory effects on the initiation and proliferation of numerous cancer cell lines, was used in the majority of those experimental studies.^15^^,^^16^^,^^18^^,^^19^

To date, contrary to the well-researched impacts of some dietary factors, the potential impact of vitamin K in preventing lung cancer is not well-established. One prospective study from Germany, which included 24,340 participants during a median follow-up of 10.2 years, reported an inverse association between total vitamin K intake and the incidence of lung cancer.^21^ Yet, evidence is lacking from Asian populations. Herein, we used the data from a large population-based prospective cohort study from Japan to investigate the association between the intake of total vitamin K and the risk of lung cancer.

## METHODS

### Study population and baseline covariates

The Japanese Ministry of Education, Sports, and Science funded the Japan Collaborative Cohort Study for Evaluation of Cancer Risk (JACC Study). A total of 110,585 middle-aged (40–79 years) Japanese individuals including 46,395 men and 64,190 women from 45 towns across Japan participated in the JACC Study baseline evaluation between 1988 and 1990. The JACC Study design and population were described elsewhere.^22^

A self-administered questionnaire that includes a 40-item food frequency questionnaire (FFQ) was used to gather baseline lifestyle and participant information, such as demographic data, family history of cancer, alcohol consumption, smoking, exercise, nutrition, and other factors. Cancer follow-up registries were available for 65,042 residents in 24 areas of the total 45 areas of the JACC study which were in the Kanto, Kinki, Hokkaido, Tohoku, Chugoku, Chubu, and Kyushu districts.

Participants with a history of cancer (*n* = 1,604) and those who did not have dietary data including vitamin K intake (*n* = 21,272) were excluded from the current investigation. Finally, we included 42,166 eligible individuals (16,341 men and 25,825 women) for the analysis (eFigure 1).

### Dietary FFQ

The normal consumption frequency for 40 food items over the previous 12 months was inquired by selecting from five kinds of frequency without defining the size of portion: seldom, 1–2 times per month, 1–2 times per week, 3–4 times per week, and nearly every day.^22^ These frequencies were converted into weekly consumption ratings of 0, 0.38, 1.5, 3.5, and 7.0. To establish the quantity of vitamin K in each food, the Japan Food Tables (5th Edition) were used to calculate dietary intakes of vitamin K. These values were multiplied by the participant’s consumption of every food item in the FFQ, then added together all of them. The portion size for each food was determined and the FFQ intakes were validated in a validation study involving 89 subjects using the weighted dietary record method, in which the subjects recorded their dietary intake for 3 consecutive days (weekdays) every 3 months for a year as a reference standard. Between the FFQ and the weighted dietary record, the Spearman rank correlation value for vitamin K intakes was 0.40. The mean intake from the FFQ was 180 (standard deviation [SD], 64) µg/day whereas from the weighed dietary record was 279 (SD, 104) µg/day.^23^

### Cancer incidence surveillance

Cancer incidence was determined using population-based cancer registries or checking major hospital records in the area. The incident cancer diagnosis was coded in accordance with the tenth revision of the International Classification of Disease, 10th revision (ICD-10) code C330–C349. The follow-up survey on the incidence and mortality of various cancers was conducted until the end of 2009; however, it ended earlier in some areas, by the end of 1994, 1997, 1999, 2000, 2002, 2003, 2006, and 2008 in one, four, one, one, eight, one, two, and two areas, respectively. Eight of 430 lung cancer cases (1.9%) were verified using death certificate only (DCO), and the remaining 98.1% were confirmed using cancer registries and records. During the study period, those who left the research region were classified as censored instances.

### Statistical analysis

Vitamin K intakes were modeled as quartiles of categorical groups in the main analysis. Categorical and continuous risk factors of lung cancer and participants’ characteristics were shown as numbers (percentage) and mean (SD), respectively. Analysis of variance and χ2-tests were used to determine the significance of variations in percentage and means of individuals’ characteristics and known lung cancer risk factors among quartiles of vitamin K intakes. Cox’s proportional hazard model, which was adjusted for age, area of residence, and sex, was used to analyze the hazard ratios (HRs) and 95% confidence intervals (CIs) of lung cancer for quartiles of energy-adjusted vitamin K consumption. We further adjusted for traditional risk factors of lung cancer in the second model, before we adjust further for dietary factors in the third model. Thus, in the final, fully adjusted analyses, we adjusted for age, sex, area of residence, alcohol intake (current, ex or never drinker), body mass index (BMI) (less than 18, 18 to <25, or ≥25 kg/m^2^), family history of cancer (with or without family history), mental stress (high or relatively high, normal, or low), smoking status (current, former, or never smoker), sports hours (never, 1–2 hours per week, 3–4 hours per week, or ≥5 hours per week), walking hours (never, <30 min per day, 30–60 min per day, or >60 min per day), educational years (<18 or ≥19 years), quartiles of energy intake, and energy-adjusted quartiles of vitamin A, vitamin C, and β-cryptoxanthin intakes. We used the residual method for calculating energy adjustment as described elsewhere.^24^ We further performed several sensitivity analyses stratifying by covariates, including sex, smoking status (never or ever smokers), age (less than 65 or ≥65 years), BMI (less than 18, 18 to <25, or ≥25 kg/m^2^), and alcohol intake (never or ever drinkers) and tested for interactions between vitamin K consumption quartiles and the dichotomized stratifying factors. Tests for linear trends were conducted to assess the dose-response associations between vitamin K intake groups and the risk of lung cancer incidence. Two-tailed statistical tests with *P* less than 0.05 regarded as statistically significant were applied using SAS statistical package (version 9.4; SAS Institute, Cary, NC, USA).

## RESULTS

Based on the sex-specific quartile intakes of vitamin K, Table 1 illustrates the sex-specific mean values and prevalence of lung cancer’s risk factor at the baseline. Males and females in the highest quartile of vitamin K intake were older, with longer sleep duration, and larger intakes of energy, vitamin A, vitamin C, and β-cryptoxanthin. They were also more likely to participate in sports, but less likely to drink alcohol, have a family history of cancer, or have high levels of perceived mental stress than those in the lowest quartile of vitamin K intake (Table 1).

**Table 1.** Sex-specific participants’ baseline characteristics according to quartiles of energy-adjusted dietary vitamin K consumption among Japanese adults

Characteristics	Energy-adjusted dietary vitamin K intake				*P* for trend

	Q1 (Low)	Q2	Q3	Q4 (High)	
Men					
Amount, µg/day	101.2 (33.7)	145.6 (33.2)	198.8 (36.5)	276.7 (40.7)	—
Subjects, *n*	4,085	4,085	4,086	4,085	—
Age, years	53.4 (9.2)	55.7 (10.1)	57.0 (10.2)	58.8 (10.1)	<0.0001
BMI, kg/m^2^	22.8 (2.8)	22.7 (2.8)	22.7 (2.8)	22.6 (2.7)	0.002
Family history of cancer, %	7.1	3.9	4.1	2.6	<0.0001
Current smokers, %	56.8	52.4	50.2	46.6	<0.0001
Current drinkers, %	79.4	75.8	72.5	70.5	<0.0001
Walking ≥1 hour/day, %	42.9	42.1	45.4	46.6	<0.0001
Sports ≥5 hour/week, %	4.5	5.7	6.4	8.3	<0.0001
Sleep duration, hours/day	7.4 (1.1)	7.4 (1.0)	7.4 (1.1)	7.5 (1.1)	<0.0001
Higher education, %	43.5	46.3	46.0	46.3	<0.0001
High perceived stress, %	13.1	11.7	10.7	11.3	<0.0001
Energy, Kcal/day	1,757 (478)	1,649 (476)	1,708 (477)	1,748 (465)	0.35
Total vegetable intake, g/day	67 (132)	138 (213)	239 (282)	502 (345)	<0.0001
Vitamin A intake, µg/day	758 (624)	930 (656)	1,167 (772)	1,447 (893)	<0.0001
Vitamin C intake, mg/day	87 (39)	110 (40)	131 (42)	160 (45)	<0.0001
β-cryptoxanthin intake, µg/day	442 (377)	544 (396)	624 (404)	736 (438)	<0.0001
Women					
Amount, µg/day	113.4 (35.3)	157.2 (33.1)	209.6 (37.8)	277.4 (37.9)	—
Subjects, *n*	6,456	6,456	6,457	6,456	—
Age, years	54.9 (9.8)	56.0 (10.0)	56.7 (9.9)	58.6 (9.7)	<0.0001
BMI, kg/m^2^	22.9 (3.2)	22.8 (3.0)	22.8 (3.1)	22.8 (3.1)	0.17
Family history of cancer, %	8.5	4.3	3.5	2.3	<0.0001
Current smokers, %	6.2	4.6	3.9	3.9	0.08
Current drinkers, %	24.9	23.9	22.6	21.6	<0.0001
Walking ≥1 hour/day, %	44.5	45.2	46.5	47.5	<0.0001
Sports ≥5 hour/week, %	3.1	3.0	4.2	5.6	<0.0001
Sleep duration, hours/day	7.0 (1.0)	7.0 (1.0)	7.0 (1.0)	7.1 (1.1)	<0.0001
Higher education, %	38.7	40.9	42.7	40.1	<0.0001
High perceived stress, %	11.7	10.4	10.1	9.9	<0.0001
Energy, Kcal/day	1,422 (380)	1,349 (334)	1,405 (353)	1,440 (333)	<0.0001
Total vegetable intake, g/day	110 (192)	201 (262)	322 (319)	563 (331)	<0.0001
Vitamin A intake, µg/day	836 (772)	987 (755)	1,169 (769)	1,454 (924)	<0.0001
Vitamin C intake, mg/day	104 (40)	123 (40)	141 (40)	166 (42)	<0.0001
β-cryptoxanthin intake, µg/day	617 (420)	698 (417)	755 (412)	855 (425)	<0.0001

BMI, body mass index.

Means (standard deviations) or percentages were presented.

During a total of 564,127 person-years of follow-up, 430 cases (308 men and 122 women) were newly diagnosed with lung cancer. In both minimally and fully adjusted models, higher dietary vitamin K consumption was linked to a decreased risk of lung cancer. The fully adjusted multivariable HR of lung cancer in the highest versus lowest quartiles of vitamin K intake was 0.67 (95% CI, 0.46–0.96; *P* for trend = 0.010). The gender of the subjects modified the observed link between vitamin K consumption and lung cancer risk (*P* for interaction = 0.012). The multivariable HRs of lung cancer in the highest versus lowest quartiles of vitamin K intake were 0.62 (95% CI, 0.40–0.96; *P* for trend = 0.016) in men and 0.82 (95% CI, 0.42–1.61; *P* for trend = 0.390) in women (Table 2).

**Table 2.** Hazard ratios (HRs) and 95% confidence intervals (CIs) of lung cancer based on quartiles of vitamin K consumption among Japanese adults

Overall	Energy-adjusted dietary vitamin K intake				*P* for trend

	Q1	Q2	Q3	Q4	
Person-years	136,337	141,447	142,580	143,764	
Number of cases	96	121	112	101	
Model 1	1.00	1.05 (0.80–1.38)	0.92 (0.70–1.21)	0.72 (0.54–0.95)	0.006
Model 2	1.00	1.06 (0.80–1.38)	0.94 (0.71–1.24)	0.74 (0.56–0.99)	0.015
Model 3	1.00	1.07 (0.80–1.42)	0.91 (0.66–1.24)	0.67 (0.46–0.96)	0.010
Men					
Person-years	53,974	55,481	54,990	55,118	
Number of cases	68	86	84	70	
Model 1	1.00	1.00 (0.72–1.38)	0.92 (0.67–1.28)	0.65 (0.46–0.91)	0.005
Model 2	1.00	1.00 (0.72–1.38)	0.95 (0.69–1.32)	0.67 (0.47–0.95)	0.012
Model 3	1.00	1.03 (0.73–1.45)	0.94 (0.64–1.37)	0.62 (0.40–0.96)	0.016
Women					
Person-years	82,363	85,966	87,589	88,646	
Number of cases	28	35	28	31	
Model 1	1.00	1.12 (0.68–1.84)	0.86 (0.50–1.45)	0.86 (0.51–1.45)	0.370
Model 2	1.00	1.12 (0.68–1.85)	0.86 (0.50–1.46)	0.87 (0.51–1.47)	0.400
Model 3	1.00	1.13 (0.67–1.92)	0.83 (0.46–1.52)	0.82 (0.42–1.61)	0.390
*P* for interaction = 0.012					

Model 1 adjusted for age (continuous) and area of residence (categories) in addition to sex in the combined analysis.

Model 2 adjusted further for alcohol intake (current, ex or never drinker), BMI (<18, 18 to <25, or ≥25 kg/m^2^), family history of cancer (with or without family history), mental stress (high or relatively high, normal, or low), smoking status (current, former, or never smoker), sports hours (never, 1–2 hours per week, 3–4 hours per week, ≥5 hours per week), walking hours (never, <30 min per day, 30–60 min per day, or >60 min per day), educational years (<18 or ≥19 years).

Model 3 adjusted further for quartiles of energy (categories), quartiles of energy-adjusted vitamin A, vitamin C, and β-cryptoxanthin intakes (categories).

Although there was no interaction with the smoking status (*P* for interaction = 0.30), a stronger association was observed among ever smokers for the highest versus lowest quartiles 0.57 (95% CI, 0.36–0.91; *P* for trend = 0.006) than never smokers 0.79 (95% CI, 0.40–1.55; *P* for trend = 0.370) (Table 3). As shown in eTable 1, there were no effect modifications for the observed lower risk of lung cancer with higher vitamin K intake by age, BMI, or alcohol intake status.

**Table 3.** Hazard ratios (HRs) and 95% confidence intervals (CIs) of lung cancer based on quartiles of vitamin K consumption stratified by smoking status among men and women combined

	Energy-adjusted dietary vitamin K intake				*P* for trend	1-SD increment of energy-adjusted vitamin K intake

	Q1	Q2	Q3	Q4		
Never smokers						
Person-years	82,122	87,173	90,670	91,807	—	—
Number of cases	28	34	31	33	—	—
Model 1	1.00	1.06 (0.64–1.75)	0.89 (0.53–1.49)	0.85 (0.51–1.42)	0.390	0.91 (0.76–1.09)
Model 2	1.00	1.05 (0.63–1.74)	0.87 (0.52–1.46)	0.83 (0.50–1.40)	0.360	0.90 (0.75–1.08)
Model 3	1.00	1.08 (0.64–1.84)	0.87 (0.48–1.57)	0.79 (0.40–1.55)	0.370	0.86 (0.67–1.10)
Ever smokers						
Person-years	48,646	48,292	45,998	45,043	—	—
Number of cases	65	85	79	62	—	—
Model 1	1.00	1.05 (0.76–1.46)	0.96 (0.69–1.34)	0.64 (0.45–0.92)	0.006	0.87 (0.77–0.98)
Model 2	1.00	1.04 (0.75–1.45)	0.94 (0.67–1.32)	0.63 (0.44–0.91)	0.004	0.86 (0.76–0.97)
Model 3	1.00	1.06 (0.75–1.50)	0.92 (0.63–1.35)	0.57 (0.36–0.91)	0.006	0.82 (0.70–0.97)
*P* for interaction = 0.300						

Model 1 adjusted for age (continuous) and area of residence (categories) in addition to sex in the combined analysis.

Model 2 adjusted further for alcohol intake (current, ex or never drinker), BMI (<18, 18 to <25, or ≥25 kg/m^2^), family history of cancer (with or without family history), mental stress (high or relatively high, normal, or low), smoking status (current, former, or never smoker), sports hours (never, 1–2 hours per week, 3–4 hours per week, ≥5 hours per week), walking hours (never, <30 min per day, 30–60 min per day, or >60 min per day), educational years (<18 or ≥19 years).

Model 3 adjusted further for quartiles of energy (categories), quartiles of energy-adjusted vitamin A, vitamin C, and β-cryptoxanthin intakes (categories).

## DISCUSSION

Higher consumption of vitamin K was related to a decreased risk of lung cancer in a dose-response pattern found in this large prospective cohort research of 16,341 men and 25,825 women with a 14.6-year median follow-up. The inverse association between vitamin K intake and risk of lung cancer was statistically significant in men but not women, stronger in ever than never smokers, and did not vary by age, BMI, or alcohol intake status of the participants.

The findings on the link between dietary vitamin K consumption and cancer risk are rudimental and unclear. Several studies have investigated the associations between vitamin K intake and the risk of cancers, including prostate cancer,^25^^,^^26^ breast cancer,^27^ pancreatic cancer,^28^ and total cancer.^21^ A study of 24,340 participants from Heidelberg with a median follow-up of 10.2 years reported an inverse association between vitamin K and the risk of lung cancer incidence and mortality. The multivariable HRs in the highest (≥46 µg per day in men and ≥42 µg per day in women) versus lowest (<26 µg per day in men and <23 µg per day in women) quartiles of menaquinones were 0.38 (95% CI, 0.20–0.71; *P* for trend = 0.002) for lung cancer incidence and 0.41 (95% CI, 0.19–0.92; *P* for trend = 0.020) for lung cancer mortality. The corresponding results for phylloquinone were 1.19 (95% CI, 0.63–2.21; *P* for trend = 0.020) and 1.08 (95% CI, 0.50–2.32; *P* for trend = 0.900), respectively. In the same study, the sex-specific analysis was conducted only for total cancer incidence, and dietary menaquinones intake was shown to be inversely related to total cancer incidence in males but not in females.

The differential effect of vitamin K by sex might be attributed to the variation in the histological type of lung cancer between men and women. In a Japanese case-control study conducted in the Miyagi Cancer Center Hospital, the proportions of adenocarcinoma, small cell carcinoma, and squamous cell carcinoma cases were 40.8%, 11.5%, and 32.7% among men versus 72.4%, 5.2%, and 7.8% among women.^29^ The most common histologic type of lung cancer among smokers is squamous cell carcinoma, while adenocarcinomas are common among non-smokers.^30^ This gender disparity in histologic type distribution is assumed to reflect differences in smoking rates among Japanese lung cancer patients (55.0% for males and 16.1% for females) in 2013. Unfortunately, the histologic type of lung cancer was not ascertained in the JACC study. In vitro studies indicated that vitamin K can inhibit oxidant-induced carcinogenesis,^31^^–^^34^ a process which is expected to be more common in men due to the higher prevalence of smoking and its related free radical generation in men than women.^35^ Accordingly, the impact of vitamin K on cancer through this process is expected to appear in men more than women.

In our study, about 81.1% of vitamin K came from the consumption of green leafy vegetables (spinach or garland chrysanthemum) and 15.1% came from the consumption of potatoes.^36^ According to a previous report from the JACC study, green leafy vegetable intake was related to a lower risk of lung cancer death among men but not women^37^; the multivariable HRs of lung cancer according to green leafy vegetable intake of ≤1 to 2 times per week, 3 to 4 times per week, and nearly every day in men were 1.00 (reference), 0.90 (95% CI, 0.71–1.14), and 0.76 (95% CI, 0.59–0.98) (*P* for trend = 0.035), while the respective HRs in women were 1.00 (reference), 1.18 (95% CI, 0.73–1.91), and 1.19 (95% CI, 0.75–1.90) (*P* for trend = 0.450). Moreover, our data showed a gender difference in the vegetable intake, with a mean of 237 (SD, 305) grams per day for men and 300 (SD, 329) grams per day for women. For the stratified analysis by smoking status, the respective HRs were 1.00 (reference), 0.98 (95% CI, 0.73–1.30), 0.80 (95% CI, 0.59–1.09) in current smoking men (*P* for trend = 0.180) and 1.00 (reference), 0.82 (95% CI, 0.51–1.33), and 0.65 (95% CI, 0.39–1.07) in ex-smoking men (*P* for trend = 0.096). The HRs among never-smoking men were not reported because of the inadequate number of lung cancer events.^37^ In the Shanghai Males’ Health Study, green leafy vegetable consumption was also linked to a lower incidence of lung cancer in 61,491 Chinese adult men; the multivariable HR was 0.72 (95% CI, 0.53–0.98; *P* for trend = 0.080) for the highest versus lowest quartiles of green-leafy vegetable intake.^38^ Another study conducted among 478,535 Europeans with a mean follow-up of 8.7 years showed that the higher consumption of vegetables and fruits was associated with a lower risk of lung cancer, especially squamous cell carcinoma among smokers; the multivariable HR of squamous cell carcinoma was 0.85 (95% CI, 0.76–0.94) with an increment of 100 g/day for calibrated total fruit and vegetable consumption among current smokers.^39^ On the other hand, a recent meta-analysis, which included four studies (one cohort and three case-control studies) on the associations between potatoes intake and lung cancer, showed that the summary effect size was 0.80 (95% CI, 0.49–1.29) for the highest intake category (quartile or tertile) versus the lowest category of total potato.^40^ The pooled odds ratio of lung cancer from two case-control studies which investigated the association between fried potato intake and the risk of the lung was 1.26 (95% CI, 0.81–1.95),^40^ while in the cohort study, the relative risks of lung cancer in the highest versus lowest quintiles of potatoes intake were 1.09 (95% CI, 0.79–1.50) among women from the Nurses’ Health Study and 1.05 (95% CI, 0.67–1.64) among men from the Health Professional follow-up Study.^40^

Several possible mechanisms might explain the observed link between dietary vitamin K consumption and the risk of lung cancer. Dietary vitamin K (phylloquinone) consumption has been known to have an inhibitory effect on systematic inflammation by reducing the level of proinflammatory cytokines, including tumor necrosis factor-α and interleukin-6,^41^ a well-known factor for driving the initiation and development of cancer. Furthermore, vitamin K acts as a cofactor in the activation of protein C, which is a protein that dependent on vitamin K, that has the potential to limit cancer cell extravasation.^42^^,^^43^

Vitamin K2 (menaquinone) has two forms; one is menaquinone-4 (MK-4), which is a product that is converted from phylloquinone in a tissue-specific manner,^44^^,^^45^ and the other is menaquinone-7 (MK-7), which could act as MK-4 and has a long-chain form with a longer half-life.^46^

Vitamin K2 has been shown to suppress nuclear factor (NF)-κB activation via inhibiting protein kinase C (PKC)- α and ε kinase activities, and inhibiting protein kinase D1 activations subsequently in cancer cells.^47^ Vitamin K3 (menadione) does not occur naturally but could be a catabolic product of phylloquinone intake in the intestine.^48^ which has strong anticancer activities by producing reactive oxygen species and disrupting the mitochondrial membrane.^16^^,^^49^ In summary, vitamin K2 and K3, which could be converted from vitamin K1 (phylloquinone),^50^ are the main natural vitamin K-driven factors to suppress the development of cancer. Total vitamin K in the diet constitutes approximately 75–90% of vitamin K1 and 25% of vitamin K2.^51^ Part (5–20%) of the ingested vitamin K is catabolized to vitamin K3 (menadione).^52^

The main strengths of our study are the prospective design with a long period of follow-up and the large sample size. Besides, we used a verified FFQ for calculating the intake of vitamin K and the cancer incidence collected through the local cancer registry or by reviewing local major hospital records, which is a reliable source of information. Further, this is one of the few studies that investigated the association between dietary vitamin K and the risk of lung cancer and is the first to show sex-specific associations between them in Asia. Finally, our several sensitivity analyses confirmed the downward trend between vitamin K intake and lung cancer across different subgroups, suggesting a high potential for extrapolating our results to more populations (eTable 1).

Our study has several limitations as a dietary epidemiology investigation without biomarker measurement. First, we could not do a separate analysis of the dietary intake of vitamin K1 and K2. However, vitamin K1 could be converted to vitamin K2 in human tissue and the actual functional amount of vitamin K2 was hard to specifically calculate. Second, the source of vitamin K was limited to the food-based intakes and in our study, the FFQ omitted the information regarding natto (fermented soybean), a major source of vitamin K in the Japanese diet. Thus, the FFQ underestimated subjects’ vitamin K consumption by at least 20% according to the dietary records-based validation research,^53^ while the Spearman rank correlation coefficient of the FFQ-estimated vitamin K intake and the DRs-estimated intake was fairly good (0.40) in term of nutritional assessment.^54^ Nevertheless, the non-differential misclassification could have attenuated the real association between the consumption of dietary vitamin K and the risk of lung cancer. Third, the intake of some nutritional factors was not adjusted in the model due to very high correlations with vitamin K, such as carotenes (*r* = 0.92), dietary fiber (*r* = 0.71), and vitamin E (*r* = 0.80). Adding these factors to the model might over-adjust the model due to the high inter-correlation. Fourth, a substantial number of participants were excluded because the cancer follow-up was conducted for 24 areas out of 45 areas of the JACC study. However, there was no substantial difference in the distribution of vitamin K intake and other participants’ characteristics between the participants included and those excluded (eTable 2). Last, there is a possibility of residual confounding by unmeasured factors remains, such as exposure to occupational carcinogens and air pollution.

In conclusion, this study indicated inverse associations between dietary intake of vitamin K and the incidence of lung cancer. Considering the food sources of vitamin K in our study, the consumption of green leafy vegetables, like spinach or garland chrysanthemum, may have a beneficial effect, especially among men.

## SUPPLEMENTARY MATERIAL

eFigure 1. Participants’ flow chart

eTable 1. Sensitivity analysis of the Hazard ratios (HRs) and 95% confidence intervals (CIs) of lung cancer according to quartiles of vitamin K intake among Japanese adults by covariates

eTable 2. Sex-specific participants’ baseline characteristics among the participants included and excluded
