# Supplementary material for: Vitamin K Intake and Risk of Lung Cancer: The Japan Collaborative Cohort Study
Source: J Epidemiol. 2023 Oct 5;33(10):536–42. doi: 10.2188/jea.JE20220063 (PMC10483107; doi:10.2188/jea.JE20220063)
Supplement: Supplementary file 1 [file je-33-536-s001.pdf]

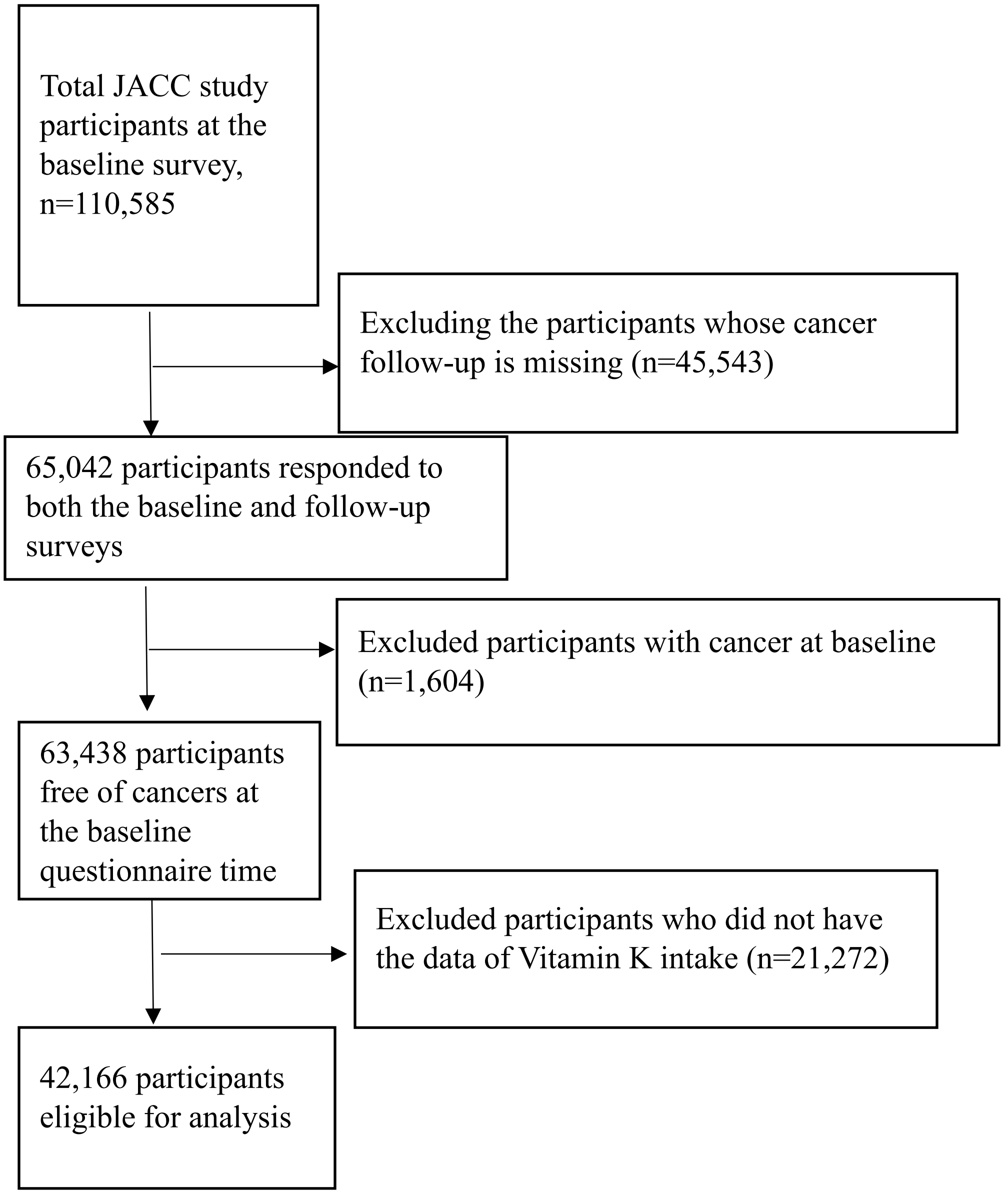

eFigure 1. Participants' flow chart

eTable 1. Sensitivity analysis of the Hazard ratios (HRs) and 95% confidence intervals (CIs) of lung cancer according to quartiles of vitamin K intake among Japanese adults by covariates

A. Sensitivity analysis by age group (<65 and ≥65 years)

	Energy-adjusted dietary vitamin K intake				*P*- trend
	Q1	Q2	Q3	Q4	
Age ≤65 years					
Person-years	121,767	120,857	119,487	113,412	
Number of cases	81	87	78	66	
Model 1	1.00	1.08 (0.80–1.46)	0.99 (0.73–1.36)	0.86 (0.62–1.20)	0.29
Model 2	1.00	1.07 (0.79–1.45)	0.96 (0.70–1.32)	0.82 (0.59–1.14)	0.17
Model 3	1.00	1.04 (0.76–1.44)	0.90 (0.63–1.30)	0.72 (0.47–1.10)	0.08
Age >65 years					
Person-years	14,570	20,590	23,093	30,352	
Number of cases	15	34	34	35	
Model 1	1.00	1.46 (0.79–2.69)	1.25 (0.68–2.31)	0.97 (0.52–1.79)	0.36
Model 2	1.00	1.46 (0.79–2.70)	1.26 (0.68–2.34)	0.96 (0.52–1.79)	0.35
Model 3	1.00	1.55 (0.82–2.95)	1.34 (0.67–2.69)	0.94 (0.43–2.06)	0.36
P-interaction = 0.42					

B. Sensitivity analysis by BMI (<18, 18 to <25, and ≥25 kg/m^2)

	Energy-adjusted dietary vitamin K intake				*P*- trend
	Q1	Q2	Q3	Q4	
BMI <18 kg/m^2					
Person-years	4,054	4,201	4,032	4,752	
Number of cases	5	4	6	6	
Model 1	1.00	0.63 (0.17–2.39)	1.00 (0.29–3.39)	0.83 (0.24–2.86)	0.96
Model 2	1.00	0.76 (0.17–3.30)	1.09 (0.30–3.92)	1.37 (0.37–5.14)	0.50
Model 3	1.00	0.51 (0.09–2.76)	0.53 (0.10–2.89)	0.70 (0.11–4.40)	0.88
18≤BMI<25 kg/m^2					
Person-years	99,062	105,333	104,729	105,856	
Number of cases	79	95	86	83	
Model 1	1.00	0.99 (0.73–1.34)	0.85 (0.62–1.16)	0.70 (0.51–0.97)	0.01
Model 2	1.00	0.99 (0.73–1.34)	0.88 (0.64–1.20)	0.72 (0.52–1.00)	0.02
Model 3	1.00	1.06 (0.77–1.46)	0.92 (0.65–1.32)	0.74 (0.49–1.12)	0.08
BMI≥25 kg/m^2					
Person-years	28,937	27,972	28,778	28,520	
Number of cases	8	18	14	10	
Model 1	1.00	2.07 (0.90–4.79)	1.50 (0.63–3.61)	0.93 (0.36–2.39)	0.50
Model 2	1.00	2.14 (0.92–4.97)	1.58 (0.65–3.81)	0.96 (0.37–2.51)	0.57
Model 3	1.00	1.62 (0.66–3.98)	1.00 (0.37–2.67)	0.46 (0.15–1.45)	0.05
P-interaction = 0.69					

C. Sensitivity analysis by alcohol intake

	Energy-adjusted dietary vitamin K intake				*P*- trend
	Q1	Q2	Q3	Q4	
Never drinker					
Person-years	66,423	71,834	75,444	75,888	
Number of cases	34	45	43	34	
Model 1	1.00	1.03 (0.66–1.62)	0.90 (0.57–1.42)	0.63 (0.38–1.02)	0.70
Model 2	1.00	1.05 (0.67–1.66)	0.93 (0.59–1.47)	0.67 (0.41–1.10)	0.06
Model 3	1.00	1.08 (0.67–1.73)	0.93 (0.55–1.56)	0.63 (0.34–1.16)	0.08
Ever drinker					
Person-years	66,750	66,286	64,071	63,768	
Number of cases	62	75	68	66	
Model 1	1.00	1.03 (0.73–1.45)	0.91 (0.64–1.29)	0.75 (0.52–1.07)	0.03
Model 2	1.00	1.06 (0.76–1.50)	0.95 (0.67–1.35)	0.78 (0.54–1.11)	0.09
Model 3	1.00	1.03 (0.72–1.49)	0.88 (0.59–1.32)	0.66 (0.42–1.05)	0.04
P-interaction = 0.31					

eTable 2. Sex-specific participants' baseline characteristics among the participants included and excluded

Characteristics	Participants included	Participants excluded
Men		
Subjects, *n*	16,341	30,054
Amount, µg /day	180.6 (74.7)	187.4 (79.9)
Age, years	56.2 (10.1)	58.3 (10.2)
BMI, kg/m²	22.7 (2.8)	22.6 (2.8)
Family history of cancer, %	4.4	2.9
Current smokers, %	51.5	50.0
Current drinkers, %	74.6	70.0
Walking ≥1 hour/day, %	44.2	33.8
Sports ≥5 hour/week, %	6.2	5.6
Sleep duration, hours/day	7.4 (1.1)	7.5 (1.2)
Higher education, %	45.5	23.4
High perceived stress, %	11.7	5.7
Energy, Kcal/day	1,716 (476)	1,834 (548)
Total vegetable intake, g/day	237 (305)	233 (302)
Vitamin A intake, µg /day	1,075 (788)	1,136 (941)
Vitamin C intake, mg/day	122 (49)	122 (51)
β-cryptoxanthin intake, µg /day	587 (419)	602 (433)
Women		
Subjects, *n*	25,825	38,365
Amount, µg /day	189.4 (71.0)	197.7 (76.2)
Age, years	56.5 (9.9)	58.8 (10.1)
BMI, kg/m²	22.9 (3.1)	23.0 (3.2)
Family history of cancer, %	4.6	2.6
Current smokers, %	4.6	4.9
Current drinkers, %	23.2	21.3
Walking ≥1 hour/day, %	45.9	7.9
Sports ≥5 hour/week, %	4.0	3.5
Sleep duration, hours/day	7.0 (1.0)	7.2 (1.2)
Higher education, %	40.6	19.4
High perceived stress, %	10.5	5.8
Energy, Kcal/day	1,404 (352)	1,464 (397)
Total vegetable intake, g/day	300 (329)	297 (331)
Vitamin A intake, µg /day	1,112 (840)	1,111 (865)
Vitamin C intake, mg/day	134 (47)	132 (49)
β-cryptoxanthin intake, µg /day	731 (427)	734 (444)

BMI, body mass index.

Means (standard deviations) or percentages were presented
